# First Free Municipal Bath Houses in America

**Published:** 1915-06

**Authors:** Francis E. Fronczak

**Affiliations:** Health Commissioner, Buffalo, N. Y.


### SPECIAL ORIGINAL ARTICLE

### First Free Municipal Bath Houses in America.

FRANCIS E. FRONCZAK, M. D.,

Health Commissioner, Buffalo, N. Y.

Read at the meeting of the American Association for Promoting Hygiene and Public Baths, City Hall, New York City, on May 11, 1915.

The city of Buffalo owns and operates, under the supervision of the Department of Health, two free public bath houses which are open every week day in the year, from 7 a. m. to 9 p. m., and on Sundays and holidays, from 7 a. m. to 10 a. m.

The baths are absolutely free, there being neither a charge for their use, nor restriction or classification of users. This free use includes an individual piece of soap, a clean towel, all the hot and cold water that can be used in the space of twenty minutes; the bather also has an opportunity to wash and dry all washable clothing.

We claim Buffalo was the first city in the world to offer facilities for washing and sterilizing clothing without expense to the user. Men living in lodging houses, those employed as sailors, men and women living in rooming houses, may come to our bath houses and be accommodated with facilities for washing and sterilizing their clothing, and take a bath in cold or warm season, and depart in clean and healthful condition.

Bath House No. 1 is located in the heart of the Italian section of the city, and Bath House No. 2 is located in our thickly populated section of the Polish district. It is needless to say that both of these bath houses are very popular. The statistics show the number of baths taken by men, women and children.

Bath House No. 1 has fourteen shower baths in individual compartments; six shower baths are provided in an open space with curtain enclosure for small boys. A bath tub is provided for infants and children too young to go under the showers; a total of 20 showers and one tub.

Bath House No. 1 was opened January 1, 1897, being the first free public bath house, not only in America, but in the world, and which not only gave a person a free bath, but a chance to wash and sterilize his clothing. The prevention of disease, by destroying vermin contained in the clothing, is, as you may well judge for yourselves, of immense value.

Bath House No. 2 was opened January 2, 1901. Here we have (14) fourteen showers for men and (9) nine showers in an open space for boys with four (4) showers for women and two porcelain bath tubs for small children and infants, also three (3) showers in an open space for girls, a total of 30 showers, 2 tubs for infants.

Both bath houses have living rooms for the keepers.

The cost of municipal free bath houses of Buffalo is as follows:

No. 1

| | |
|---|---|
| Cost of site | $ 6,500 |
| Cost of building | 8,000 |
| Cost of equipment | 300 |
| | $14,800 |

No. 2

| | |
|---|---|
| Cost of site | $ 2,600 |
| Cost of buildings and equipment | 16,365 |
| | $18,965 |

Tables Showing Patronage of Public Baths and Laundry Facilities for the Year 1914.

Free Bath House No. 1—Terrace.

| | Table of Baths Taken. | | | | Laundry used for washing and drying clothes. | | |
|---|---|---|---|---|---|---|---|
| 1914 | Men | Women | Children | Totals | Men | Wom. | Tot. |
| Jan. | 4763 | 151 | 829 | 5743 | 485 | 5 | 490 |
| Feb. | 3754 | 93 | 590 | 4437 | 434 | 6 | 440 |
| Mar. | 5221 | 120 | 804 | 6145 | 493 | 6 | 499 |
| Apr. | 5419 | 149 | 1221 | 6789 | 481 | 6 | 487 |
| May | 5640 | 254 | 2021 | 7915 | 415 | 5 | 420 |
| June | 4722 | 208 | 2363 | 7293 | 212 | 6 | 218 |
| July | 6971 | 369 | 3062 | 10402 | 352 | 8 | 360 |
| Aug. | 6899 | 442 | 2788 | 10129 | 520 | 9 | 529 |
| Sept. | 5675 | 244 | 1603 | 7522 | 530 | 6 | 536 |
| Oct. | 5144 | 168 | 1597 | 6909 | 487 | 5 | 492 |
| Nov. | 4177 | 168 | 882 | 5227 | 478 | 6 | 484 |
| Dec. | 4466 | 160 | 916 | 5542 | 600 | 5 | 605 |
| Totals | 62851 | 2526 | 18676 | 84053 | 5487 | 73 | 5560 |

Free Bath House No. 2
Woltz Avenue and Stanislaus Street.

| Table of Baths taken during the Year 1914. | | | | | Laundry used for washing and drying clothes. | | |
|---|---|---|---|---|---|---|---|
| 1914 | Men | Women | Children | Totals | Men | Wom. | Tot. |
| Jan. .... | 5687 | 523 | 883 | 7093 | 290 | 10 | 300 |
| Feb. .... | 5023 | 478 | 744 | 6245 | 216 | 8 | 224 |
| Mar. ... | 6608 | 610 | 1205 | 8423 | 250 | 10 | 260 |
| Apr. ... | 8058 | 826 | 1774 | 10658 | 205 | 9 | 214 |
| May ... | 8566 | 1105 | 2319 | 11990 | 415 | 5 | 420 |
| June ... | 9696 | 1525 | 4675 | 15896 | 94 | 9 | 103 |
| July ... | 9128 | 1932 | 8211 | 19271 | 70 | 10 | 80 |
| Aug. ... | 9113 | 1630 | 7750 | 18493 | 85 | 8 | 93 |
| Sept. ... | 7125 | 922 | 2897 | 10944 | 78 | 10 | 88 |
| Oct. ..... | 6178 | 729 | 1947 | 8854 | 101 | 8 | 109 |
| Nov. ... | 6398 | 632 | 1209 | 8239 | 173 | 8 | 181 |
| Dec. .... | 6677 | 707 | 1171 | 8555 | 234 | 10 | 244 |
| Totals .. | 88257 | 11619 | 34785 | 134661 | 2211 | 105 | 2316 |

Maintenance Bath House No. 1.

| Year 1913. | | Year 1914. | |
|---|---|---|---|
| Salaries ............ | $1600.00 | Salaries ............ | $1999.72 |
| Towels ............. | 410.28 | Towels ............ | 376.54 |
| Soap ............... | 208.82 | Soap .............. | 91.92 |
| Incidentals ......... | 34.16 | Incidentals ......... | 13.55 |
| Total ............ | $2253.26 | Total ............ | $2473.73 |
| | | Repairs ............ | 678.22 |
| | | Fuel, (Est.) ........ | 1520.00 |
| | | | $4671.95 |

Maintenance Bath House No. 2.

| Year 1913. | | Year 1914. | |
|---|---|---|---|
| Salaries ............ | $3040.00 | Salaries ............ | $3790.00 |
| Towels ............. | 597.21 | Towels ............. | 678.29 |
| Soap .............. | 203.69 | Soap .............. | 214.88 |
| Furniture .......... | 20.00 | Incidentals ......... | 86.87 |
| Incidentals ......... | 41.33 | Total ............ | $4770.04 |
| Total ............ | $3902.23 | Repairs ............ | 548.98 |
| | | Fuel ............... | 2064.50 |
| | | | $7383.52 |

There is no income of any kind from baths, as they are absolutely free in every respect.

Number of employees and their salaries:

Bath House No. 1.

| | Per annum. |
|---|---|
| Keeper | $ 900 |
| Matron | 650 |
| Asst. Keeper | 900 |

Bath House No. 2

| | |
|---|---|
| Keeper | $ 900 |
| Matron | 700 |
| Asst. Keeper | 900 |
| Two firemen, each | 1020 |

Construction.

Both bath houses are of substantial construction. The bathing compartments and the walls are of brick, painted with enamel paint; the floors of concrete, and the partitions of slate in iron frames.

The system is the ''Rain Bath'' and an inclined shower delivering water at a maximum temperature of 100 degrees under the control of the bather, as regards volume and degree of temperature.

Each alcove is divided into two parts—the dressing room and bathing room separated by partial slate partitions. The shower room has a depression in the floor six inches deep, all angles being rounded off, and supplied with waste perforations of inadequate capacity to carry off the water as fast as delivered by the shower; thus, water rises about four inches making a good foot bath—the bather stands erect and the shower strikes the body from the head down.

A system of public baths is of great importance, not only as a means of healthful living, but also as a necessity for counteracting the unsanitary conditions of the occupants of tenement and lodging houses and dwellings not provided with bath facilities. The cultivation of the habit of personal cleanliness has a favorable effect upon the character; tending to self respect and decency of life.

In most cities the lodging houses are not provided with bathing facilities. Buffalo, however, is not in this class as every one of our lodging houses is equipped with at least one shower bath. The tub bath is not recommended from a sanitary point of view in lodging houses, as they are not kept clean, and do not answer the purpose for bathing facilities in these places of abode. Statistics show that a very small percentage of persons occupying tenement houses and dwellings have access to bathrooms. The only way in which occupants of such houses can bathe is by using a tub of some kind, filled from the faucet in the kitchen or hall. It is apparent that such conditions as these do not encourage the practice of bathing. The number of rooms occupied by a family in a tenement house, or in a dwelling, is generally so small that every inch of space is occupied. Even when the occupants are willing to incur labor of carrying water from the faucet in the hall or from the kitchen, it is difficult to secure the privacy which is necessary for the bath. In many families, today, boarders are kept, owing to the economic conditions, and under such circumstances, taking a bath without a bathroom is impossible. Thousands of dollars are spent annually for public parks, playgrounds, high schools, etc., and little money is provided for public bath houses which are among the greatest assets of a city.

Active steps are being taken by the Buffalo Health Department to put at least two additional municipal bath houses in the thickly populated industrial districts of Black Rock and South Buffalo, and we have good reason to hope for early realization of our endeavors in this direction. I do not speak in this paper of baths in the various school houses, which are free in every respect, but are limited to the school children: neither is mention made of municipally supervised open air baths in Lake Erie at the foot of South Michigan Avenue, and in Niagara River, which are opened only in summer of course.

The Bulletin of the Bureau of Labor, Department of Commerce and Labor, issued a very full report on "Public Baths in the United States," written by G. W. W. Hanger in September, 1904, same being part of Bulletin No. 54 of the Bureau publication. Additional information on the subject may be found among others in the following publications:

"Public Baths and Wash Houses," by A. W. Cross, 1906.

"Modern Baths and Bath Houses," by W. P. Gerhard, 1908.

New York City Mayor's Committee on Public Baths and Public Comfort Stations—report issued in 1897.

---

**Pernicious Anaemia.** A. M. Gossage of Westminster Hospital, Med. Chron., April 1915, reports in detail several cases treated with salvarsan which he considers superior to ordinary forms of arsenic. He thinks this method may be quite as efficient as splenectomy which is dangerous, Mühsam having lost 5 of 15 cases.

